# Optimal long-term antithrombotic management of atrial fibrillation: life cycle management

**DOI:** 10.1007/s12471-018-1118-0

**Published:** 2018-05-02

**Authors:** R. Pisters, A. Elvan, H. J. G. M. Crijns, M. E. W. Hemels

**Affiliations:** 1 Department of Cardiology, Rijnstate Arnhem, Arnhem, The Netherlands; 2 Department of Cardiology, Isala Hospital Zwolle, Zwolle, The Netherlands; 3 Department of Cardiology, Maastricht University Medical Centre, Maastricht, The Netherlands; 4 Department of Cardiology, Radboud University Medical Centre Nijmegen, Nijmegen, The Netherlands

**Keywords:** Atrial fibrillation, Anticoagulation, Stroke risk, Bleeding risk, NOAC, VKA

## Abstract

Optimal antithrombotic management of atrial fibrillation equals balancing between prevention of arterial thromboembolism, predominantly ischaemic stroke, and haemorrhagic complications. Over time different antithrombotic agents and strategies have been developed. At present, non-vitamin K antagonist oral anticoagulants (NOACs) are the first-line therapy for stroke prevention in patients with non-valvular atrial fibrillation (i.e. without a mechanical valve prosthesis or rheumatic heart disease). Considering the impact of the suboptimal adoption of recommended oral anticoagulant therapy, as experienced with the previous first-line vitamin K antagonists, this review focuses on adequate use of NOACs. As such, we address the most important and clinically challenging issues in the antithrombotic life cycle management for long-term stroke prevention in atrial fibrillation.

## Introduction

Ischaemic stroke is probably the most infamous complication of non-valvular atrial fibrillation (AF). Untreated, the average annual stroke risk is 5% and exceeds 7% if clinically undetected strokes and transient ischaemic attacks (TIAs) are taken into account [1]. These observations triggered an ongoing search for the optimal antithrombotic strategy: a practical approach for maximum stroke and bleeding risk reduction. Anno 2018, this strategy consists of 1) AF documentation, 2) long-term stroke risk assessment using the CHA_2_DS_2_-VASc score (congestive heart failure, hypertension, age ≥75 years [doubled], diabetes mellitus, prior stroke [doubled], vascular disease, age 65–74 years and female sex), 3) modifiable stroke and bleeding risk factor identification and 4) appropriate initiation of a non-vitamin K antagonist oral anticoagulant (NOAC), unless contra-indicated [2].

**Figure:**
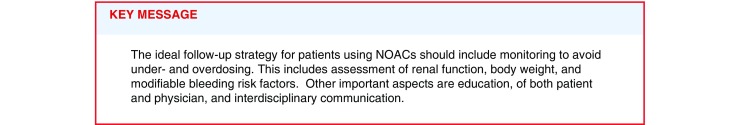

**Fig. 1:**
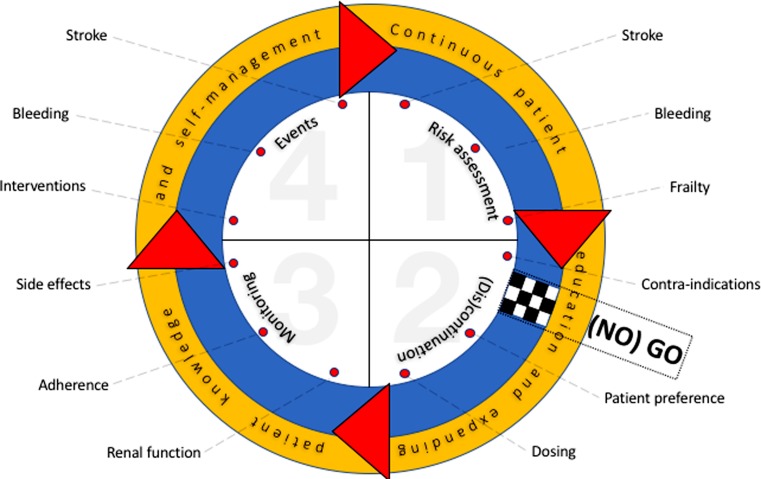
Oral anticoagulation life-cycle management in atrial fibrillation

The above-mentioned integrated AF treatment approach appears simple and straightforward and appropriate use results in a very substantial stroke and mortality reduction. However, inappropriate use or deprivation of patients from an effective strategy may lead to suboptimal clinical outcome. The latter is one specific lesson learned from the vitamin K antagonist (VKA) era [3]. This review focuses on adequate use of NOACs to prevent history from repeating itself. As such, we address the most important and clinically challenging issues in antithrombotic life cycle management for long-term stroke prevention in AF (Fig. 1).

## Initiation of long-term antithrombotic management

### AF documentation and indication for oral anticoagulation (OAC)

Given the significant antithrombotic therapeutic consequences and their potential sequelae, it all starts with unequivocal electrocardiographic documentation of AF. Next, identifying clinical AF duration and type, adequate treatment of symptoms, underlying cardiovascular and pulmonary disease and/or other triggers. However, it is worth noting that the indication for OAC is independent of the type of AF. A clinically challenging scenario is the patient with secondary AF: a paroxysmal episode only documented during a temporary, reversible disease state (e.g. thyrotoxicosis, post-operative inflammation, and infections such as pneumonia). These patients have both a higher risk of ‘future’ AF and worse long-term cardiovascular outcomes [4]. However, optimal long-term antithrombotic management of secondary AF remains unclear in the absence of randomised controlled trials (RCTs). Despite the association of OAC use with reduced long-term mortality in post-operative AF patients [4], appropriate risk assessment, timing and patient preference need to be taken into account. Currently, the same accounts for atrial sensing of asymptomatic high atrial rate episodes by implantable devices. There is supporting evidence that the majority of these patients with ‘subclinical AF’ also have an increased stroke risk [5, 6], it is still unclear whether and when they benefit from OAC, also considering the bleeding risk. OAC has to be considered in patients with two additional CHA_2_DS_2_-VASc risk factors (i.e. ≥2 in males, ≥3 in females) and an AF burden >24 h (if there are no contraindications) [7]. Lower duration may merit OAC if multiple risk factors are present.

#### Short-term stroke risk

Somewhat artificially we can differentiate a short- and long-term stroke risk. The former has a different, more ‘mechanical’ pathophysiology. Functional left atrial (appendage) standstill during AF is followed by resolution of atrial stunning upon sinus rhythm restoration [8]. This mechanism is thought to be responsible for the post-cardioversion strokes, nearly all occurring within 10 days after cardioversion [9]. This relates to the known proportional relationship between the duration of the AF episode and the time until normal atrial contraction returns [8, 10]. Similarly, there is a risk of peri-ablation left atrial appendage thrombus. The optimal management of this short-term stroke risk is a separate entity and depends on the planned procedure, i.e. cardioversion or ablation.

### Long-term stroke risk assessment

The vascular nature of AF is underlined by the high prevalence of cardiovascular co-morbidities, which represent the intrinsic long-term stroke risk. The most important independent stroke risk factors are incorporated into the CHA_2_DS_2_VASc score and its use is recommended for stroke risk assessment in AF patients [2]. Considering the increasing aging of our society it is important to emphasise that age is an extremely strong, continuous, risk factor for stroke [11] and aging potentiates other risk factors such as heart failure. Therefore, the CHA_2_DS_2_VASc score should be reassessed over time [12]. Untreated, one out of every four octogenarians with AF suffers a stroke during their remaining lifetime [1]. Still, across various health care settings studies demonstrated age, frailty and dementia to be some of the strongest independent predictors of withholding anticoagulation in the eldest elderly [13–15]. However, in these patients the key question is not whether, but how we should organise optimal anticoagulation.

### Major bleeding risk assessment

The downside of any antithrombotic drug is an increase of patients’ intrinsic risk of bleeding. It therefore makes sense to identify bleeding risk factors. Practical bleeding risk scores, such as HASBLED [16], assist in particular with identifying modifiable risk factors. Thereby minimising the risk of major bleeding during OAC treatment. However, we should keep in mind that the available risk scores fail to capture important frailty aspects in the eldest elderly with AF [17] and, similarly in elderly with venous thromboembolism [13]. Here, previous reports demonstrated that age itself proved to be a strong determinant of risk. Especially the eldest elderly (85+) appear to have a substantial increased risk of major bleeding [18, 19]. This enhanced bleeding risk also applies for patients with a poor quality of anticoagulation (i.e. time in therapeutic range [TTR] <65%, patients spent a median 14%, 71%, and 15% of time below, within, and above the intended therapeutic range, respectively) [20].

### Choosing anticoagulation: shared decision making

According to the current European Society of Cardiology/European Heart Rhythm Association (ESC/EHRA) guidelines men and women with a CHA_2_DS_2_VASc score of 2 and 3, respectively, clearly benefit from OAC. In case of a CHA_2_DS_2_VASc score of 1 in men and 2 in women, OAC is likely to provide net clinical benefit and individual characteristics and patients’ preferences should be considered [2]. However, a primary care study among 260 AF patients aged 70–85 years that used decision analysis regarding antithrombotic treatment preference showed that approximately half of the patients with a preference for OAC did not receive it [21]. On the other hand, 47% of patients were not being prescribed warfarin although the results of their decision analysis suggested they wanted to be. A researcher-administered questionnaire study using a fictional AF patient and pictograms to display the 10-year risk of stroke showed that 100% of the elderly chose OAC over no treatment. However, when the necessity of many tablets with multiple blood tests, alcohol restrictions and an increasing risk of intracranial haemorrhage was introduced, the choice for anticoagulation dropped. A daily tablet corresponds to 94%, an intracranial haemorrhage risk of 0.1%/year corresponds to 99% and 0 units of alcohol/day corresponds to 89% who would still choose OAC [22]. Although most physicians will acknowledge its importance, practical and evidence-based methods of shared decision making in AF patients are an unmet need (Table 1).

**Table 1:** Preconditions for effective shared decision making

Motivation	Communication	Infrastructure
Willingness	Stroke risk and impact	ICT support
Social support	Bleeding risk and impact	Substitution of care
Cognitive capability	On a patient level	Time

### Choosing anticoagulation: aspirin is no suitable alternative

The downside of any antithrombotic drug is the increase of the patients’ intrinsic bleeding risk. Undeniably, fear for bleeding has caused physicians to choose aspirin over VKA for perceived lower bleeding risk. However, it is important to realise that there is no relationship between a drug’s ability to prevent stroke and its potential to harm through bleeding (safety paradox). In fact, irrespective of age, aspirin use in AF patients is associated with a similar rate of major bleeding events compared with VKA but with significantly inferior stroke protection [23, 24]. This also holds true for the eldest elderly (*n* = 366 ≥ 85 years): aspirin vs. apixaban, 4.9% vs. 4.7% major bleeding events and 7.5% vs. 1.0% strokes, respectively, per year in one study [19]. Furthermore, the combination of aspirin with clopidogrel also provides inferior stroke protection in AF patients compared with VKA [25]. Finally, OAC is the only antithrombotic regimen that reduces all-cause death [26].

Thus, even in AF patients with a low stroke risk (i.e. CHA_2_DS_2_VASc score <2 in men and <3 in women), aspirin is not a suitable alternative, it warrants either no antithrombotic therapy or OAC [2]. However, we should keep in mind that these patients may require aspirin for the prevention of other atherosclerotic complications.

### Choosing anticoagulation: NOACs

The collaborative evidence from multiple, large clinical trials randomising elderly AF patients to a NOAC or VKA consistently demonstrated at least similar efficacy and safety, but significantly less intracranial haemorrhage with all NOACs compared with VKA [2]. In addition, compared with VKAs, NOACs are much easier to use with a rapid onset and offset (comparable with low-molecular-weight heparins), standard daily dosing (no need for frequent routine blood tests) and limited drug interactions. Therefore, NOACs are the current first-line oral anticoagulant in the absence of a contra-indication [2].

NOACs are only contra-indicated in the infrequent case of, usually rheumatic, moderate/severe mitral stenosis, end-stage renal disease, or mechanical heart valves. These subgroups were excluded from the NOAC trials. Furthermore, the thromboembolic pathophysiology in both rheumatic valve disease [1] and mechanical heart valves is significantly different. For now, in the case of the above-mentioned groups VKAs are indicated.

**Fig. 2:**
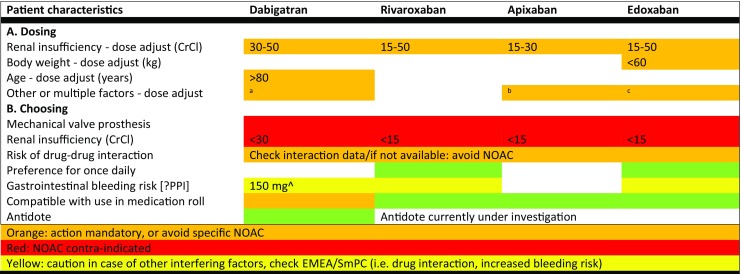
Dosing (**A**) and choosing (**B**) a NOAC in atrial fibrillation. The proposals voiced here are not based on head to head comparisons of the four NOACs but are derived from European labelling (EMA, and related SmPCs) and current ESC guidelines on AF. *AF* atrial fibrillation*, BID* twice daily,* CrCl* creatinine clearance, *EMA* European Medicines Agency, *ESC* European Society of Cardiology, *NOAC* non-vitamin K antagonist oral anticoagulant, *P-gp* P-glycoprotein, *SmPC* summary of product characteristics, ^a^Verapamil, ^b^If 2 out of 3: age ≥80 years, weight ≤60 kg, serum creatinine ≥133 µmol/L ^c^Strong P‑gp inhibitor (excluding verapamil), check EMEA/SmPC, ^d^PPI Consider proton pump inhibitor, ^e^Preference for 110 mg BID in case of gastritis/oesophagitis

In the absence of head-to-head randomised controlled trials there is no superior NOAC. The value of these drugs lies in the significant benefits over VKAs and aspirin as discussed above. Still, nuances between the respective landmark trials (e.g. baseline stroke risk factors, age, the occurrence of side effects), practical aspects (e.g. once vs. twice daily, tablet/capsule size, blister packaging, antidote) and pharmacokinetics (e.g. reduced renal function, drug interactions) allow for choice (Fig. 2). Furthermore, the prescribing physician’s experience with and preference for specific NOACs should play a role in this choice.

Of much greater importance is choosing the appropriate NOAC dose in order to replicate RCT results in daily clinical practice and pursue optimal stroke protection (Fig. 2). This seems logical, but ‘real-world’ data consistently show label-discordant dosing, in particular an inappropriately lower dose [27–32]. Although the reasons remain speculative, it could be because of a perceived increased risk of bleeding. However, physicians should be aware of the safety paradox: inappropriate low dosing results in potentially preventable strokes [33, 34], not significantly fewer major bleeding events [33, 35]. In order to appropriately dose NOACs the relevant criteria must be checked. Renal function is important for all NOACs, but information about recent renal function was lacking in a third of the AF patients in a large international prospective NOAC registry [32]. This calls for nationwide prospective registries, such as DUTCH-AF (Project number 848050006, *ZonMw-programma Goed Gebruik Geneesmiddelen*).

## Aspects of successful long-term antithrombotic management

Long-term antithrombotic management does not stop following the OAC and NOAC prescription. The ideal follow-up strategy for patients using NOACs is unclear. It should include monitoring to avoid under- and overdosing. In addition, periodic assessment of modifiable bleeding risk factors and renal function seems appropriate because it matters for safety and/or dose recommendation for all NOACs. Another important aspect is education, of both patient and physician, and interdisciplinary communication. There appears to be room for improvement [36] as suggested by the outcome from a recent cluster RCT investigating an educational intervention program [37]. Of the 371 control group patients with a long-term indication for OAC only 5.9% was aware of medical emergencies (e.g. melena or signs suggestive of stroke). Knowledge was also scarce regarding what analgesics are safest to use (15.4%), what to do after a missed dose (19.9%), when to inform others about their OAC use (23.9%) and target international normalised ratio (45.5%) [37]. On the upside, the educational intervention significantly increased the knowledge level which sustained up to 24 months in that study [37].

### Antithrombotic management following major bleeding and stroke

As pointed out by Maikranz et al. [37] it appears that very few patients with long-term OAC know how to act in case of unexpected major bleeding events. However, it is important to realise (and communicate) that these major events occur despite optimal preventive measures and to discuss with the patient how to act.

A predominantly upper gastrointestinal bleeding is the most frequent major bleeding complication in patients using long-term antithrombotic treatment, both aspirin and OAC [38]. Although gastrointestinal bleeding in itself is often non-fatal, it is a prognostic marker as reflected by a death rate of 30–35% in two years following the index event [39, 40]. Restarting OAC following a gastrointestinal bleeding is the only regimen associated with both a very significant reduction of all-cause mortality [39–43] and stroke. Although the risk of major bleeding increases with reinitiating OAC, the risk of a recurrent gastrointestinal bleed does not [39, 40], except in patients with end-stage renal disease [43]. In case of repetitive gastrointestinal bleeding, quality of life also plays an important role in the shared decision to whether or not restart OAC therapy. Furthermore, it is important to take the potential mechanism into account. For instance, esophagogastroduodenoscopy confirmed ulcer healing before initiation of OAC significantly reduces the rate of major bleeding [44]. Also, good (>65%) opposed to poor (<65%) TTR did not increase the risk of a recurrent ulcer bleeding but provided better stroke prevention [42]. The optimal time window of reinitiating OAC following gastrointestinal bleeding remains unclear. Qureshi et al. found that restarting OAC seven compared with thirty days after a gastrointestinal bleeding did not increase the risk of major bleeding but was associated with lower mortality and stroke rates [39].

Regarding the most feared and fatal bleeding complication, intracranial haemorrhage, a Danish nationwide study revealed that AF patients who survived the initial six weeks following an intracranial haemorrhage had a substantial reduction of all-cause mortality and stroke compared with no OAC during one-year follow-up (hazard ratio 0.55, confidence interval 0.39–0.78) [45]. A Swedish nationwide study showed that of the surviving 1,454 patients of a first intracranial haemorrhage under OAC, 10.4% resumed OAC within three and 21.2% within twelve months [46]. Oral anticoagulants should probably not be resumed in patients with a lobar intracerebral haemorrhage caused by cerebral amyloid angiopathy, given the high risk (3–14% within one year) of recurrent intracranial haemorrhage [47]. Although RCTs answering the question whether and when to resume antithrombotic medication in patients who suffered an intracranial haemorrhage are lacking, one is currently recruiting in the Netherlands (APACHE AF trial) (Table 2). Hence, resuming antithrombotic medication should be a multidisciplinary decision, involving a cardiologist, neurologist and a geriatric specialist or general practitioner [48].

**Table 2:** Registered randomised clinical trials on long-term antithrombotic management of atrial fibrillation

Trial number	Start	End	Title	Acronym	*n*
NCT02928133	04.2014	05.2018	NOACs for Atrial Tachyarrhythmias in Congenital Heart Disease	NOTE	300
NCT01994265	10.2014	05.2018	Cognitive Impairment Related to Atrial Fibrillation Prevention Trial	GIRAF	200
NCT02941978	12.2015	05.2018	Motivational Interviewing to Support Oral AntiCoagulation Adherence in Patients With Non-valvular Atrial Fibrillation	MISOAC-AF	1000
NCT02690649	01.2016	06.2018	Keep it SIMPLE: Improving Anti-Coagulation Medication Adherence		250
NTR5532	01.2016	08.2018	Management of Atrial fibriLLation INcluding tailoring of anticoagulation in patients from primary care	ALL-IN	1000
NCT03174093	06.2017	11.2018	Mhealth Application for anTicoagulation Care in Atrial	MATCh AFib	200
NCT02889562	09.2016	12.2018	Apixaban Versus Warfarin for the Management of Post-operative Atrial Fibrillation		56
NCT02666157	01.2016	12.2018	Comparison of Efficacy and Safety Among Dabigatran, Rivaroxaban, and Apixaban in Non-Valvular Atrial Fibrillation	DARING-AF	3672
NCT02933697	04.2017	04.2019	Compare Apixaban and Vitamin-K Antagonists in Patients With Atrial Fibrillation (AF) and End-Stage Kidney Disease (ESKD)	AXADIA	222
NCT02618577	02.2016	05.2019	Non-vitamin K Antagonist Oral Anticoagulants in Patients With Atrial High Rate Episodes	NOAH	3400
NCT02942407	12.2016	05.2019	Trial to Evaluate Anticoagulation Therapy in Hemodialysis Patients With Atrial Fibrillation	RENAL-AF	762
NCT03126214	05.2017	09.2019	Improving Stroke Prevention in Atrial Fibrillation Through Pharmacist Prescribing	PIAAF Rx	370
NCT02998905	04.2017	01.2020	NOACs for Stroke Prevention in Patients With Atrial Fibrillation and Previous ICH (NASPAF-ICH)	NASPAF-ICH	100
NCT02426944	04.2015	05.2020	Left Atrial Appendage Closure vs. Novel Anticoagulation Agents in Atrial Fibrillation	PRAGUE-17	400
NCT02961348	02.2017	12.2020	TIMING of Oral Anticoagulant Therapy in Acute Ischemic Stroke With Atrial Fibrillation		3000
NCT02387229	03.2015	02.2021	Blinded Randomized Trial of Anticoagulation to Prevent Ischemic Stroke and Neurocognitive Impairment in AF	BRAIN-AF	6396
NCT03061006	04.2017	04.2021	Impact of Anticoagulation Therapy on the Cognitive Decline and Dementia in Patients With Non-Valvular Atrial	CAF	120
NCT01938248	05.2015	04.2021	Apixaban for the Reduction of Thrombo-Embolism in Patients With Device-Detected Sub-Clinical Atrial Fibrillation	ARTESiA	4000
NCT03148457	07.2017	07.2021	Early Versus Late Initiation of Direct Oral Anticoagulants in Post-ischaemic Stroke Patients With Atrial fibrillatioN (ELAN): an International, Multicentre, Randomised-controlled, Two-arm, Assessor-blinded Trial	ELAN	2000
NCT03021928	06.2017	08.2021	Optimal Delay Time to Initiate Anticoagulation After Ischemic Stroke in Atrial Fibrillation	START	1000
NCT03129490	04.2017	09.2021	The Danish Non-vitamin K Antagonist Oral Anticoagulation Study in Patients With Atrial Fibrillation	DANNOAC-AF	11000
NCT02168829	01.2016	12.2021	Optimal Anticoagulation for Higher Risk Patients Post-Catheter Ablation for Atrial Fibrillation Trial	OCEAN	1452
NCT02886962	01.2017	01.2023	Oral Anticoagulation in Haemodialysis Patients	AVKDIAL	6396
NCT02928497	02.2017	12.2023	Assessment of the WATCHMAN™ Device in Patients Unsuitable for Oral Anticoagulation	ASAP-TOO	888
NCT02830152	05.2017	05.2030	Prevention of Stroke by Left Atrial Appendage Closure in Atrial Fibrillation Patients After Intracerebral Hemorrhage		750
NCT02565693	09.2014		Apixaban Versus Antiplatelet Drugs or no Antithrombotic Drugs After Anticoagulation-associated Intracerebral Haemorrhage in Patients With Atrial Fibrillation	APACHE-AF	100

Early stroke recurrence of cardioembolic origin is as high as 14% within two weeks. OAC substantially reduces this risk. Even, or perhaps especially, the eldest elderly benefit as Appelros et al. demonstrated a reduced all-cause mortality and stroke rate in nonagenarians (between 90 and 100 years of age) [49]. Initiation of OAC monotherapy, which outperforms low-molecular-weight heparin alone or followed by OAC, 4–14 days following the index stroke opposed to outside this window provides the best protection against recurrent stroke [50]. Perhaps beside the use of the National Institutes of Health stroke severity scale [2], the novel ALESSA score might assist in deciding upon early initiation or re-initiation [51].

**Fig. 3:**
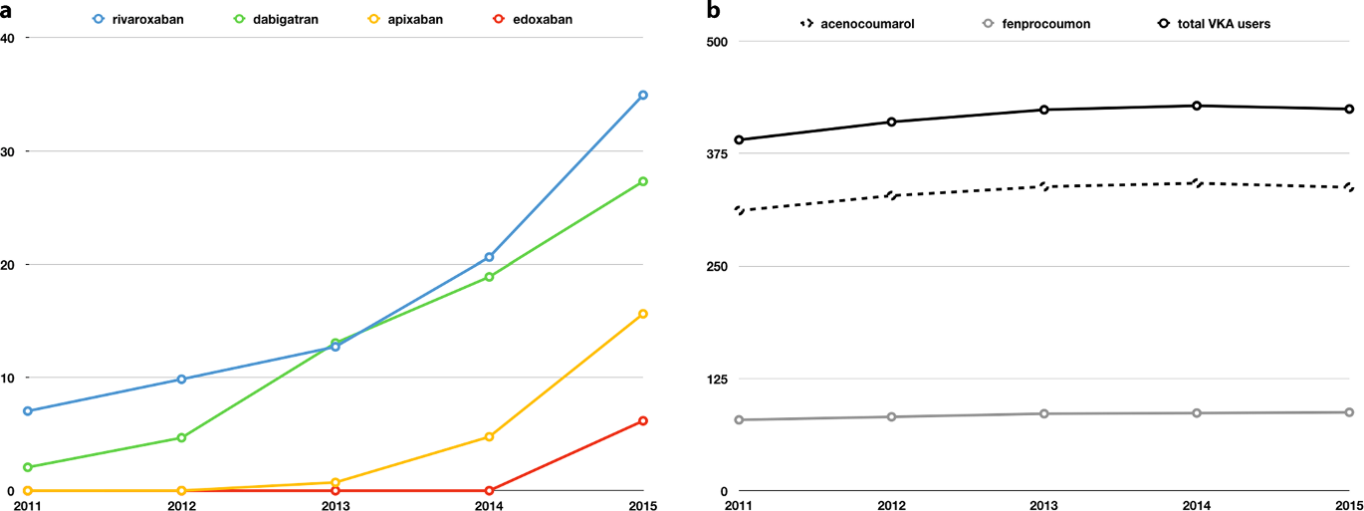
Prescription rates of non-vitamin K antagonist oral anticoagulants (**a**) and vitamin K antagonists (**b**) in the Netherlands

**Fig. 4:**
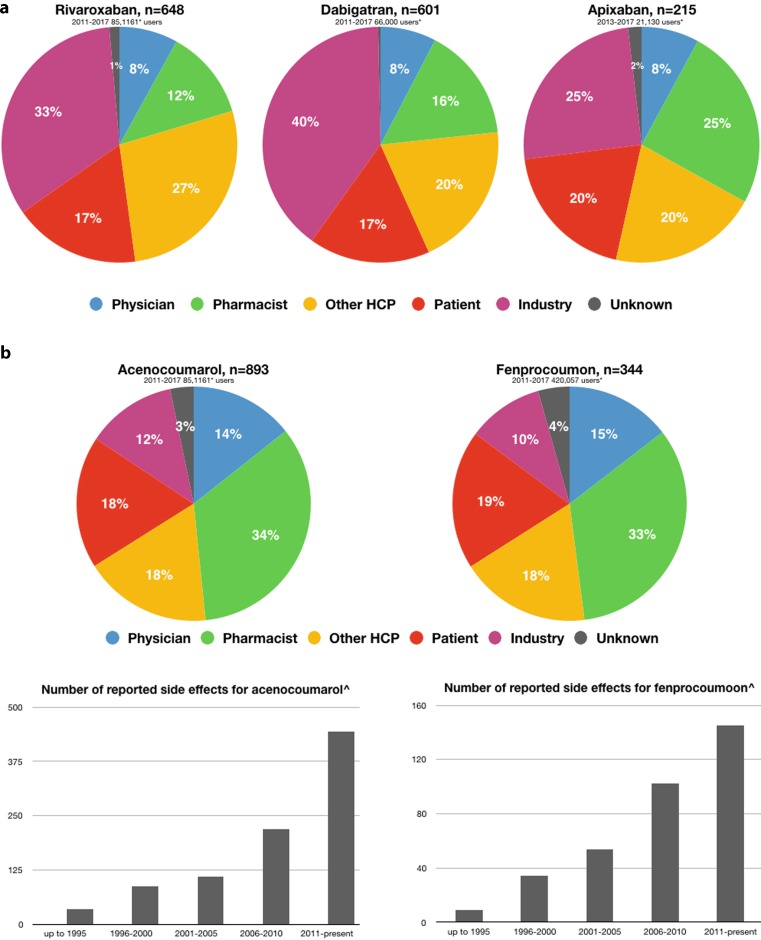
Reporterd side-effects of non-vitamin K antagonist oral anticoagulants (**a**) and vitamin K antagonists (**b**) in the Netherlands (**a** Source: www.lareb.nl; updated 4.7.17. At this moment in time too few side-effects regarding edoxaban were reported to portrait a breakdown., **b** Source: www.gipdatabank.nl; updated 22.11.16)

Side effects could trigger incorrect or non-use of OAC leading to a vicious circle. It therefore deserves special attention during follow-up. However, it seems as if a magnifying glass is being held over the side effects of NOACs as reflected by the registration in the Netherlands (Lareb). Interestingly, the industry filed most (35%) of the reports followed by patients (17–20%) and pharmacists (16%), whereas physicians (8%) provided the least of the reports. In the Netherlands, rivaroxaban and dabigatran became available in 2011, followed by apixaban (2013) and edoxaban (2015) (Fig. 3a). Since then, the Netherlands pharmacovigilance centre Lareb received a total of 1,487 (609 were severe) side effect notifications among 84,047 NOAC users. A NOAC specific breakdown is displayed in Fig. 4a. During the same time period (2011-present) among the >400,000 VKA users (Fig. 3b) Lareb received a total of 1,237 (366 were severe) side effect reports (Fig. 4b). Perhaps, this provides food for thought on how we can optimise the monitoring and reporting of long-term side effects in the near future. Furthermore, results of real-life studies and registries are instrumental in understanding how new therapies find their way in daily clinical practice and for the identification of pitfalls.

Several ongoing and future trials will also provide answers on current knowledge gaps (Table 2).

## End of long-term antithrombotic management

The rate of OAC use only declines despite an increasing risk of stroke over time [11, 52]. Until recently this was partly driven by the fact that there was no suitable alternative for VKA treatment. Many of the previously discussed aspects such as patient education, frailty, reduced life expectancy, stroke, major bleeding or drug specific side effects contribute to the withholding or permanent discontinuation of OAC. In the contemporary RCTs permanent OAC discontinuation was roughly 20% in two years, with a higher rate of cessation in the first year of follow-up. Permanent discontinuation occurred significantly more with dabigatran and significantly less with apixaban compared with warfarin and aspirin [24, 53, 54]. In ‘real life’, permanent discontinuation can be as low as 20% in five years, even in the VKA-naive eldest elderly [55]. Despite the previously discussed superior reduction of all-cause mortality and stroke, in selected patients OAC is permanently discontinued after a careful shared decision process, similar to deactivating the defibrillator capacity of the ICD. However, literature to support best practice on this topic is lacking.

## Conclusions

Current clinical knowledge and evidence concerning optimal long-term antithrombotic management of AF patients provide clear recommendations for the majority of patients. However, a relentless suboptimal use of OAC remains a major concern, especially in the elderly. Several ongoing RCTs aim to fill ‘the knowledge gap’ in specific patient groups or situations concerning the start, continuation or discontinuation of OAC. Still, important unmet needs are improved education, guidance on shared decision making [56], assessment and impact of frailty, and prospective registries of daily clinical practice, such as DUTCH-AF (Project number 848050006, *ZonMw-programma Goed Gebruik Geneesmiddelen*).
